# Occurrence and determinants of parental psychosocial stress and mental health disorders in parents and their children in early childhood: rationale, objectives, and design of the population-based SKKIPPI cohort study

**DOI:** 10.1007/s00127-020-02004-6

**Published:** 2020-12-18

**Authors:** J. Fricke, M. Bolster, C. Ludwig-Körner, L. Kuchinke, F. Schlensog-Schuster, P. Vienhues, T. Reinhold, A. Berghöfer, S. Roll, T. Keil

**Affiliations:** 1 Institute for Social Medicine, Epidemiology and Health Economics, Charité-Universitätsmedizin Berlin, Luisenstr. 57, 10117 Berlin, Germany; 2 International Psychoanalytic University, Berlin, Germany; 3 Department of Child and Adolescent Psychiatry, Psychotherapy and Psychosomatics, University of Leipzig, Leipzig, Germany; 4 Department of Psychiatry, Psychosomatics and Psychotherapy, Diakonissenkrankenhaus Flensburg, Flensburg, Germany; 5 Institute of Clinical Epidemiology and Biometry, University of Wuerzburg, Wuerzburg, Germany; 6 State Institute of Health, Bavarian Health and Food Safety Authority, Bad Kissingen, Germany

**Keywords:** Early childhood, Parental mental health, Regulatory problems, Cohort study, Service utilisation

## Abstract

**Purpose:**

The postnatal period is a vulnerable time for parents and children but epidemiological and health care utilisation data for Germany on parental mental health during early childhood is scarce. This protocol describes the rationale, aim and study design of a population-based cohort study to assess the occurrence and determinants of psychosocial stress and mental health disorders, as well as the use and cost of health care and social services in early childhood.

**Methods:**

As part of the collaborative SKKIPPI project, we will contact a random sample of 30,000 infants listed in the residents’ registration offices of three German towns and we expect to include 6,000 mother–child pairs. Both parents are invited to fill out an online screening questionnaire. Mothers with indications of psychosocial stress will be interviewed to assess mental health disorders, regulatory problems of their children, as well as health care and social services utilisation, with a follow-up assessment after 6 months.

**Results:**

After description of sociodemographic and health data, we will analyse occurrences, patterns, and potential determinants (maternal age, social status, household factors, migration status etc.) of psychosocial stress and mental health disorders in the mothers and their children in early childhood.

**Conclusions:**

Our study will identify potential risk and protective factors for postnatal mental health and health care utilization of psychosocially burdened families. This will help to improve prevention and treatment strategies to strengthen the parent–child relationship, to reduce persisting vulnerability of children, and to improve health care and social services.

**Trial registration:**

The study has been registered in the German Clinical Trial Registry on February 8th 2019 (DRKS-ID: DRKS00016653).

## Rationale

Psychosocial stress in parents with young children is not only a burden for their own mental health but may also negatively affect the children’s short and long-term mental well-being [1]. Epidemiological studies on maternal mental health disorders in the postnatal period have been almost exclusively focused on postnatal depression and anxiety disorders [2–4]. For postnatal depression, prevalence estimates revealed large variations worldwide [3, 4]. A meta-analysis based only on studies using the Edinburgh Postnatal Depression Scale (EPDS)—a self-report screening instrument for postnatal depression [5]—assessed a global prevalence of almost 18% [4]. An international review including also studies using psychiatric interviews revealed prevalences ranging from 4 to 64% [3]. According to another systematic review, the prevalence of self-reported postnatal anxiety symptoms was 15% and of clinically diagnosed anxiety disorders around 10% [2]. The few German studies assessing prevalence data on postnatal maternal mental health disorders revealed estimates between 3 and 6% for postnatal depression and around 11% for postnatal anxiety [6–8]. Ongoing research is focusing especially on postnatal depression or anxiety disorders, but there are less studies assessing prevalence data of other postnatal mental health problems like obsessive–compulsive disorders [9, 10] or parental burnout, a concept that has only recently been suggested and investigated [11, 12]. One of the few studies in this field reported that 10.4% of the parents with at least one child at home belonged to an average parental burnout category and 2.1% of the parents to a high parental burnout category, but the sample included parents with children of all ages [13].

Various stressors for an elevated risk of postnatal depression and anxiety disorders have been identified. These include a history of previous depression, single marital status, lack of social support, illnesses and sleep problems of the child, socio-economic status, or an unplanned/unwanted pregnancy [14–17]. Longitudinal studies from Sweden and the Netherlands found that parental stress in early parenthood was associated with anxiety during pregnancy as well as with postnatal depressive symptoms [18, 19]. Another risk factor for pre- and postnatal depression may be the experience of childhood trauma, like childhood sexual abuse [20]. Protective factors that seem to be associated with maternal health disorders are supportive couple's relationships [21] and breastfeeding [22], but are less well studied than risk factors.

As the first postnatal months are an important period for the child’s physical and mental development, studies have focused on how this development of the child is affected by poor maternal mental health [1, 23–25]. One negative consequence in early childhood seems to be an elevated vulnerability for regulatory problems, meaning problems of feeding, sleeping or excessive crying [26–28]. Population-based studies, which collected data on regulatory problems, assessed rates up to 21% within the first 2 years of life [29, 30]. A recent study from Germany reported rates of 10.1% for excessive crying, of 36.4% for feeding problems and of 12.2% for sleeping problems [31], with the first two regulatory problems seeming to be associated with maternal anxiety and the latter with maternal depression [28]. There are also studies indicating bidirectional effects of parental and children's mental health [32, 33]. Children with regulatory problems also seem to have an elevated risk for later psychopathological symptoms in the childhood period [34, 35]. But children of parents with mental health problems are not only at risk in the childhood period, indeed they seems to be at an elevated risk to develop poor mental health outcomes at some point in their lifetime, a concept called ‘trans-generational transmission’ [36–38].

Reasons for developmental problems of children with parents showing poor mental health in early childhood can in part be explained by an impaired parent-infant-relationship [39, 40]. Thus, therapeutic interventions should not only address parents or children separately, but must also focus on the parent-infant relationship [41]. One promising approach are specialized parent-infant psychotherapies [42, 43]. In Germany (like in other countries), there are out- and inpatient health care structures specialized in offering treatment for perinatal mental health disorders, for example mother-baby-units within psychiatric hospitals [44, 45] or specialized psychotherapeutic outpatient clinics [46, 47], offering parent–child psychotherapy. Another example are early support programs beginning directly after birth in the maternity ward, like the Babylotse Plus program for psychosocially burdened mothers and their infants [48]. To allow better access to psychosocial support for pregnant women and families with children aged 0–3 years, a public Early Childhood Intervention (ECI) program has been implemented in Germany in 2006 [49]. The program is open to all pregnant women and families with young children, with additional support for families in need. Services include home visits by health care professionals or volunteers and more intensive parenting programs to develop secure infant-parent attachment [49].

Even though there are specialized structures and programmes, knowledge of postnatal mental health service provision for parents seems to be insufficient. In Germany, a study by the National Centre of Early Prevention revealed a clear difference between educational groups in knowledge and use of assistance programmes supporting families with psychosocial burden [50]. A systematic review found that women experience barriers in seeking and receiving psychological and psychosocial interventions, for example for postnatal depression [51]. One obvious barrier seems to be the stigma associated with mental health disorders [51, 52]. There is little information on health care costs associated with postnatal mental health problems, because health economic data in this area has rarely been evaluated. A study from UK calculated total lifetime costs of perinatal depression to be £ 75,728 per woman with this disorder, most of it related to adverse impacts on the child [53].

Overall, more population-based epidemiological data on mental health in early childhood as well as information on health and social care service utilisation are needed to clarify the situation in Germany.

The collaborative German *SKKIPPI* project would like to make a contribution to this and includes an observational population-based cohort study as well as two interventional studies (https://skkippi.ipu-berlin.de/). The SKKIPPI intervention studies are randomized controlled trials (RCT) that will evaluate the efficacy of a specific parent-infant/toddler psychotherapy (6 week program according to a specific SKKIPPI Study Treatment Manual) compared to care-as-usual in mothers with mental health disorders and in mothers of children with regulatory problems, respectively. The two RCTs will be performed in Berlin (both RCTs), Flensburg (only RCT for mother with mental health disorders) and Leipzig (only RCT for mothers with children with regulatory problems), so within the same cities as the cohort study, but they will be described separately. Participants of the cohort study, which qualify for the interventional studies, are invited to take part in the RCTs. If they agree to participate, they will be excluded from the follow up assessment of the cohort study. SKKIPPI is performed within and funded by the German Health Care Innovation Fund, aiming at improving quality and integration of health care [54]. The present article focuses on the rationale, objectives, and design of the observational population-based cohort study as part of the SKKIPPI project.

The primary aim of the SKKIPPI cohort study is to assess the occurrence and course of psychosocial stress and mental health disorders in parents and their children in early childhood in three German regions. Furthermore, potential risk and protective factors as well as the use of health care and social service related to these disorders will be examined.

## Methods

### Objectives of the study

The objectives of the SKKIPPI cohort study are:

i. To assess the occurrence of psychosocial stress and mental health disorders in parents and their children in early childhood.
ii. To assess protective and risk factors for psychosocial stress and mental health disorders in parents and their children.
iii. To assess the health care and social services utilisation and associated costs of parents with psychosocial stress and mental health disorders and their children [services based on German Social Security Code V (health care service: outpatient and inpatient sector) and services based on German Social Security Code VIII, IX, XII (Early Childhood Intervention program sector)].

### Design

The SKKIPPI cohort study is a prospective observational population-based cohort study with a two-step screening design.

### Sample characteristics

Parents with children aged up to 12 months (at the time the sample is drawn) will be recruited from three German cities: Berlin (3,600,000 inhabitants), Leipzig (560,000 inhabitants), and Flensburg (86,000 inhabitants). In these areas combined, annually almost 49,000 children have been born in recent years [55–57]. Out of all registered children aged 12 months and younger, a random sample of 30,000 contact addresses of the children's parents will be drawn from the residents' registration offices including 25,200 from Berlin, 4250 from Leipzig, and 550 from Flensburg, according to the proportion of births. We chose this approach to reach as many parents in the respective regions as possible. Parents are invited by postal letter to participate in the study. The focus of our study is on the mothers (biological and adoptive mothers), but fathers are encouraged to participate as well, to gain insights into their psychosocial stressors after birth and to identify further research needs. With an expected response of about 20% based on another German population study [58], about 6000 mother–child pairs are expected to participate in our study. For the screening step 1 mothers with children up to 2 and a half years are included. If the mother qualifies for follow-up assessment the children may be up to 3 years of age.

The inclusion criteria are:

- Mother or father (natural or adoptive) with a child up to the age of 12 months at the time of sampling, registered in Berlin, Leipzig, or Flensburg
- Written or online informed consent
- Parental age of at least 18 years
- Participants are able to understand the German, English, Turkish, or Arabic language

### Procedures

**Fig. 1:**
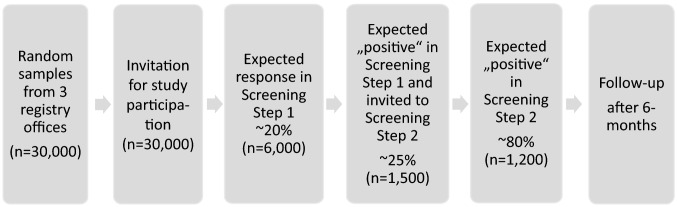
Flowchart SKKIPPI Cohort Study

To identify participants at risk, a two-step screening design will be implemented (Fig. 1). 30,000 potential participants receive an invitation letter, primarily addressing the mother, while fathers are also encouraged to participate in the screening step 1. In this first step, parental symptoms of psychological stress and signs of regulatory problems in the children will be assessed via online questionnaire.

While both mothers and fathers can participate in the first step, for the second step, only mothers will be included. As mothers are usually the main reference persons of the children in the first years of life, a significantly higher participation of them is expected. For this reason, we decided to assess mental health problems only in mothers and their children. Mothers who screened “positive” in the first step, meaning achieved at least 10 points in the online questionnaire, will enter the second step of the screening for a more in-depth assessment, where specially trained study staff will conduct phone interviews for mental health disorders (adapted version from the MINI [59]). In addition, questions on depressive symptoms, symptoms of parental burnout, symptoms of regulatory problems of the children, questions on childhood trauma of the parents, and detailed data on kind and frequency of health and social care utilisation will be collected (via telephone interview or online questionnaire).

After 6 months, a follow-up assessment will be performed with all mothers with a current mental health disorder (based on the MINI interview [59]) or a positive outcome in the Edinburgh Postnatal Depression Scale (EPDS) [5] or Parental Burnout Assessment questionnaire (PBA) [12] within the screening step 2 (“positive” screening step 2). The same data as in the screening step 2 stage will be collected, with additional data about the use of health care services and official support services in the last 6 months. We estimated that 80% of the participants would take part in the follow-up assessment.

### Endpoints

The primary endpoints of the cohort study are:

i. *For mothers*: (i) presence of at least one current mental health disorder (based on the M.I.N.I interview [59]). (ii) presence of parental burnout symptoms (defined as score of ≥ 76 in the Parental Burnout Assessment questionnaire [12]). (iii) Presence of depressive symptoms (defined as an Edinburgh Postnatal Depression Scale (EPDS) score of 10–12 points (minor symptoms) or ≥ 13 points (major symptoms) versus 0–9 points (no symptoms) [5]
ii. *For children*: presence of problems concerning crying, feeding and sleeping (so-called regulatory problems) (defined as a total score of ≥ 1.85 in the questionnaire on crying, feeding and sleeping disorders [60]). Subscales (crying, feeding and sleeping) will also be evaluated individually.

Secondary endpoints include:

- *For mothers*: presence of the following current mental health disorders: major depressive episode, major depressive disorder, manic and hypomanic episodes, panic disorder, agoraphobia, social anxiety disorder (social phobia), obsessive–compulsive disorder, posttraumatic stress disorder, alcohol use disorder, substance use disorder (non-alcohol), Anorexia nervosa, Bulimia nervosa, binge-eating disorder, generalized anxiety disorders.
- *For mothers and children*: health care and social services utilisation and costs: medical consultations, utilisation of midwifery care/physical therapy/speech therapy/occupational therapy, acute and rehabilitation hospital stays, utilisation of Early Childhood Intervention program components etc..

Additionally, the course and patterns of the observed disorders and problems will be evaluated by a follow-up assessment after 6 months. Potential risk and protective factors such as sociodemographic characteristics, marital, family, health, and work related stressors, critical life events, and pre-existing mental health disorders will be examined. If fathers agree to participate in the screening step 1, we will have the information of the latter also for the fathers.

### Instruments

#### Screening step 1

In the screening step 1, study participants fill out a short online-questionnaire. The questionnaire was developed with the aim of having a high sensitivity to detect potential mental health disorders and psychosocial stress. By implementing a two-step screening design we hope to reach a high sensitivity and high specificity, allowing an accurate early detection of stress or mental health problems in parents and their children. There are existing screening instruments like the Babylotse-Plus screening form [61] or the Heidelberg Stress scale [62], but they showed either a high sensitivity and low specificity [61] or only a moderate sensitivity [62].

The questionnaire for the screening step 1 (self-developed and adapted parts) contains questions regarding the parent’s health status: symptoms of depression and/or anxiety, obsessive–compulsive problems, abuse/addiction to alcohol/drugs, eating problems, lack of impulse control or mood swings, pre-existing mental health disorders. Questions regarding the child include the assessment of signs of crying, sleeping or feeding problems.

In addition, questions on sociodemographic characteristics, stress related to relationships, family and work, lack of social support and critical life events related to the pregnancy and child (for example an unplanned pregnancy or severe illness of the child) will be assessed. To reduce selection bias, the questionnaire was kept as long as necessary but as short as possible and in simple language, to achieve a high participation rate especially in those with a lower educational level or non-native German speakers. Otherwise, particularly those at risk for psychosocial stress and mental health disorders might be under-represented in the study. Therefore, either short questionnaires such as the Patient Health Questionnaire 4 [63] or individual questions from existing questionnaires were selected or adapted (the Crying, Feeding, Sleeping Questionnaire [60], the Zohar-Fineberg Obsessive Compulsive Screen [64], the Postpartum Bonding questionnaire [65], and the KID 0–3 questionnaire [66]). We developed a scoring system with points ranging from 0–5 for each question. The scores were discussed with a panel of experts from psychology, paediatrics, health sciences, and data sciences, taken into account cut-offs of included questionnaires. We piloted the questionnaire and adapted it as indicated (e.g. adding or rewording of response options). The scope of this pilot study was to test if the questionnaire was comprehensible, appropriate and feasible for the participants. Cut-offs were defined consensus based in order to identify a maximum of mothers with possible disorders, but the data from pilot study did not allow to conduct an in-depth analysis to calculate sensitivity or specificity. All mothers with an elevated score are invited to the screening step 2. This score was adjusted later on because the results of the first participants showed that too many mothers were included in the screening step 2 showing no current mental health problems. To increase the response among the major groups of parents with a migration background, all study documents of the screening step 1 were translated to English, Turkish, and Arabic.

#### Screening step 2

The screening step 2 (Table 1) includes the following questionnaires:

- Adapted version of the M.I.N.I. Version 7.02, a structured interview based on DSM-V and ICD-10 [59]. Suicidal and psychotic symptoms will only be assessed using an abbreviated version and are, therefore, not diagnostically interpretable. Modules on antisocial personality disorder and bipolar disorders were not considered, but major depressive episodes and manic/hypomanic episodes will be assessed separately.
- Parental Burnout Assessment (PBA) [12].
- Questionnaire on crying, feeding and sleeping disorders [60].
- Edinburgh Postnatal Depression Scale (EPDS) [5, 67].
- Questions on childhood trauma of parents [66].
- Questionnaires on the use of health care and psychosocial services (self-developed).

**Table 1:** Screening steps and instruments

Screening step	Instrument
Screening step 1	Self-developed questionnaire on parental psychosocial stress symptoms and signs of regulatory problems in the children
Screening step 2	MINI [59]
Screening step 2	Parental burnout assessment [12]
Screening step 2	Edinburgh postnatal depression scale [5, 67]
Screening step	Questionnaire on crying, feeding and sleeping [60]
Screening step 2	Questions on childhood trauma of the parents [66]
Screening step 2	Self-developed questionnaire on health care and psychosocial service utilization

All interviewers will receive an intensive training based on Standard Operating Procedures (SOP). This training is focusing especially on the acquisition of interviews techniques to assess accurately mental health disorders using the M.I.N.I. interview [59]. After the formal training, each interviewer will do test interviews for several weeks under continuous supervision. Regular supervision of the interviewers will take place.

### Statistical analysis

In the three recruitment areas of the cohort study (Berlin, Leipzig and Flensburg) almost 49,000 children have been born annually in recent years. A random sample of 30,000 children is drawn by the residents' registration offices, and their parents are invited to take part in the study. The response is estimated to be 20%; therefore, about 6000 mother–child pairs are expected to participate in the study. Assuming a prevalence of mental health problems in postnatal mothers of about 20% [8], a similar prevalence of regulatory problems in their children [68] and a considerable overlap of both problems, about 1,500 of the estimated 6,000 mother–child-pairs are expected to be included into the screening step 2 (Fig. 1). In the screening step 2, we estimate that about 1,200 participating mother–child-pairs with maternal mental health disorders and/or symptoms of regulatory problems in the child will be identified. For an occurrence of 20% of mental health disorders (1200 of 6000), a two-sided exact Clopper-Pearson 95%-confidence interval (95% CI) of 6000 participants has a width of ± 1% (i.e., 95% CI [19%; 21%]). This is considered sufficiently precise.

All data will be analysed descriptively. Results are presented in the form of means, standard deviations, medians and quartiles for continuous data, and frequencies and percentages for categorical data. Results (including occurrences) are reported with two-sided 95% CIs. Influencing factors for the presence of psychosocial stress or mental health disorders are analysed using logistic regression yielding odds ratios (OR) with 95% CIs and *p* values. Influencing factors or group comparisons for continuous variables (e.g. costs) will be analysed by linear regression or analysis of covariance (ANCOVA), yielding regression coefficients or means with 95% CIs and *p* values. All CIs and *p* values are two-sided and are interpreted exploratively (there is no formal significance level). Missing values are not replaced. All analyses are carried out with the respective available data (full analysis set).

For the cost analysis, the monetary valuation of the utilization of services is carried out by multiplying the frequency of utilization and corresponding standardized cost rates.

## Preliminary results

By mid-March 2020 29,516 of the potential study participants received at least an invitation letter and at least one reminder. Overall, 4719 mothers and 889 fathers completed the screening step 1. Three participants were adoptive parents and three participants did indicate to be other caregivers (for example stepparents). So far, either the mother or the father or both parents of 4866 children (16.5%) have completed the screening step 1 questionnaire. All persons gave their informed consent prior to their inclusion in the study.

4472 participants came from Berlin, 75 from Flensburg, 933 from Leipzig and the remaining participants were living elsewhere at the time of the screening step 1. 811 participants were younger than 30 years, 4779 between 30 and 49 years and 21 participants 50 years or older (three values missing). 860 participants did indicate to be born outside Germany, 272 to be single parents and 2351 participants having more than one child.

Overall, 1139 mothers (24.1%) and 154 fathers (17.3%) screened so far “positive” in the screening step 1. As described above, only mothers with a “positive” screening step 1 are invited to the screening step 2.

## Discussion

This is the first study in Germany using a large population-based random sample from local registry offices to assess psychosocial stress and mental health disorders in parents and their children in early childhood. We chose a cohort design for the present study to assess longitudinal data after birth and to have a look at how psychosocial stress and mental health disorders are developing over a 6 month observation period.

### Strengths and potential limitations

It is important to detect parents with an elevated stress level at a very early stage, to plan preventive strategies to reduce the risk for developing mental health disorders in both parents and children. We developed a short screening instrument for psychosocial stress in early childhood. It includes known relevant stressors associated with postnatal mental health disorders and should be helpful to identify vulnerable parents and their children in the future. The simple screening questionnaire is accessible online and can be filled out by the invited parents (estimated time 10–15 min). We discussed the questionnaires with experts from a multidisciplinary background and adapted them after a piloting phase.

For the screening step 2, we needed instruments to assess the predefined outcomes in mothers under risk as well as in their children. To assess the occurrence of mental health disorders a psychiatric interview is considered the gold standard. We, therefore, decided to use an adapted version of the MINI [59], because it is a short structured and validated interview. A recent study from Sweden investigated the acceptability of the MINI in a primary care setting and concluded that the MINI interview was well accepted by patients and general practitioners [69]. Using the MINI not only occurrence rates of postnatal depression or anxiety, but also the occurrence of other mental health disorders—like obsessive–compulsive disorders or eating disorders—will be assessed. This will add more information regarding these mental health disorders in mothers with children in the early childhood period. One limitation of the MINI is that it is a complex interview. The interviewers in our study will, therefore, receive an extensive training before performing interviews for the screening step 2. Another limitation is that a psychiatric interview may be potentially distressing for participants who until that moment may not have had any contact with psychiatric assessments. A sensitive introduction to the interview is, therefore, essential and part of the interviewer training. The additional assessment of EPDS scores will allow us to compare our study with data from other international studies and to assess mild depressive symptoms that do not meet the diagnostic criteria for major depression disorder. Using the EPDS Scores, we will also observe how depressive symptoms are developing over the 6 month observation period.

For the assessment of symptoms of regulatory problems of the child, we will use the questionnaire on crying, feeding and sleeping disorders [60], which is the only self-reported questionnaire currently available for this kind of problems. We expect that mothers with an elevated stress level or a mental health disorder may report more often that their children are showing signs of regulatory problems. Therefore, the occurrence of this kind of problems may be over-estimated, because we have only information based on the information of the mother. We also decided to evaluate the rather new hypothetical construct of parental burnout and we will use a specific questionnaire that is currently being tested in different countries and different languages worldwide (The International Investigation of Parental Burnout [12]).

Besides assessing epidemiological data, we are interested in analyzing the actual health care and social service utilization for this understudied subpopulation in Germany. For this purpose, we developed a questionnaire containing questions regarding health care and social services utilization of the participating families. The knowledge of specific utilisation behaviour by different subgroups may help to better address health care and social services needs in the future. We would expect that the variety of available services is complex and that it may discourage particularly less educated parents from making use of it.

One limitation of our study is the generalizability of our sample, which is coming from only three registry offices. Our results may be considered representative for these regions but not for the rest of Germany, especially not for rural areas. All recruitment regions are urban areas of different size including regions from Northern as well as Eastern Germany. The participants may be possibly considered as being representative for these regions to some extent, but certainly not for the rest of Germany. Interpreting the statistical results, we will carefully evaluate possible types of bias including sampling bias considering our recruitment strategy.

We decided to invite only parents with children already born to participate in this study (instead of inviting women while still pregnant). Otherwise, we would not have been able to use the registry offices for sampling. Using this approach, we hope to reach as many participants as possible in the respective regions. However, we know that we will have limited information on some risk factors during pregnancy. We decided to evaluate only mothers and their children in the screening step 2, because mothers are usually the main reference persons of the children in the first years of life. However, future studies should consider assessing more information about fathers and their special situation and needs within the first years after birth. We excluded participants under 18 years due to ethical and organisational reasons, such as acquiring written informed consents of their parents as well, i.e., the grandparents of the index child. Study participation might be lower among parents with lower educational levels as compared to higher educational levels, as seen in many other studies [70–73]. Therefore, we are expecting an underrepresentation of socially disadvantaged parents in our study population. Where possible, we adapted the study documents keeping them as short as possible and using simple language to decrease the selection bias. On the other hand, potential participants are much more likely to take part in a study when the subject is salient to the participants’ life [73], which we expect for our observational cohort study.

Our hypothesis is that mothers with an elevated level of psychosocial stress are often showing mental health symptoms. In addition, we expect that their children are showing signs of regulatory problems. If we are looking at patterns, we expect to find the mental health problems to remain relatively stable if the mothers or children are not receiving support – for example by social and health care institutions. And we expect to replicate already known determinants of mental health problems as well as further possible factors.

### Prospects

Although there is no legal obligation, nearly all children in Germany are regularly seen in the first years after birth by primary care pediatricians, for example for the routine well-baby/child visits [74]. However, their parents are not. One possibility to overcome this problem may be to screen parents also regularly during the routine well-baby/child visits. A systematic review provided some positive evidence for a screening in such settings [75]. The American Academy of Pediatrics already recommends that primary care pediatricians screen mothers within the first 6 months after births in the course of the regular pediatrician visits [76]. Currently, no screening for postnatal stress or mental health disorders is regularly foreseen by the health care or social services sector in Germany.

## Conclusions

Epidemiological data on psychosocial stress and mental health disorders in parents and their children in early childhood in Germany is limited. Population-based data is urgently needed and will close knowledge gaps of the specific situation of mothers and their children during this early phase of life in Germany and meet their future needs in the health care and social services sector.
